# Selective Autophagy in *Drosophila*

**DOI:** 10.1155/2012/146767

**Published:** 2012-04-10

**Authors:** Ioannis P. Nezis

**Affiliations:** ^1^Department of Biochemistry, Institute for Cancer Research, Oslo University Hospital, Montebello, 0310 Oslo, Norway; ^2^Centre for Cancer Biomedicine, Faculty of Medicine, University of Oslo, Montebello, 0310 Oslo, Norway; ^3^Laboratory of Cell Biology, Department of Biological Applications and Technologies, University of Ioannina, 45110 Ioannina, Greece

## Abstract

Autophagy is an evolutionarily conserved process of cellular self-eating and is a major pathway for degradation of cytoplasmic material by the lysosomal machinery. Autophagy functions as a cellular response in nutrient starvation, but it is also associated with the removal of protein aggregates and damaged organelles and therefore plays an important role in the quality control of proteins and organelles. Although it was initially believed that autophagy occurs randomly in the cell, during the last years, there is growing evidence that sequestration and degradation of cytoplasmic material by autophagy can be selective. Given the important role of autophagy and selective autophagy in several disease-related processes such as neurodegeneration, infections, and tumorigenesis, it is important to understand the molecular mechanisms of selective autophagy, especially at the organismal level. *Drosophila* is an excellent genetically modifiable model organism exhibiting high conservation in the autophagic machinery. However, the regulation and mechanisms of selective autophagy in *Drosophila* have been largely unexplored. In this paper, I will present an overview of the current knowledge about selective autophagy in *Drosophila*.

## 1. Introduction

Macroautophagy (from hereafter referred to as autophagy) is an evolutionarily conserved process by which a portion of the cytosol and organelles are sequestered by isolation membranes called phagophores. The phagophore engulfs portions of the cytoplasm and forms a double-membrane-layered organelle called the autophagosome. The autophagosome then fuses with a lysosome and generate the autolysosome that has a single limiting membrane, where its sequestered components are degraded [1]. Autophagy serves as a cellular response in nutrient starvation, but it is also responsible for the removal of aggregated proteins, damaged organelles, and developmental remodeling and therefore plays an important role in the quality control of proteins and organelles and in cellular homeostasis [1]. Genetic inhibition of autophagy induces degeneration that resembles degeneration observed during ageing, and physiological ageing is associated with reduced autophagic activity [2]. Autophagy is implicated in neurodegeneration, infections, tumorigenesis, heart disease, liver and lung disease, myopathies, and in lysosomal storage disorders [2]. Interestingly, it has been shown that induction of autophagy can increase longevity in multiple animal species [3]. Contrary to the belief that autophagy is a nonselective process, recent evidence suggests that degradation of proteins, protein aggregates, organelles, and bacteria can be selective through adaptor proteins [4]. It is therefore important to elucidate the role of selective autophagy in normal and pathological conditions using model organisms. The fruit fly *Drosophila melanogaster* is a genetically modifiable model organism and is an excellent model for investigating the mechanisms of selective autophagy in the context of the physiology of the cell, the system, and the living organism. This paper will summarize the current knowledge about selective autophagy in *Drosophila*.

## 2. Selective Autophagy in *Drosophila*

Studies in *Drosophila* so far revealed the presence of highly conserved autophagic machinery compared to yeast and mammals [5]. *atg* (autophagy-related) genes and their regulators in *Drosophila* in many cases, in contrast to mammalian systems, have single orthologs, allowing for nonredundant genetic studies [5]. However, the regulation and mechanisms of selective autophagy have not been described in details, and there is only limited evidence for the presence of selective autophagy and autophagic cargo receptors. Additionally, cellular processes related to selective autophagy like mitophagy (selective autophagy of mitochondria), xenophagy (selective autophagy of bacteria and viruses), nucleophagy (selective autophagy of nucleus), and pexophagy (selective autophagy of peroxisomes) are largely unexplored in *Drosophila*. In the following text, I will describe what is reported so far in the literature about selective autophagy and selective autophagy-related proteins in *Drosophila.*

### 2.1. Selective Autophagy Receptors in *Drosophila*

#### 2.1.1. Ref(2)P, the *Drosophila* Homologue of the Mammalian Selective Autophagy Receptor p62/SQSTM1

In mammals, six proteins have been identified as selective autophagy receptors so far: p62/SQSTM1, NBR1, NDP52, Nix, optineurin, and Stbd1 [4, 6, 7]. These proteins contain a LIR/LRS (LC3-interacting region/LC3 recognition sequence) motif and have been shown to interact with the autophagosomal membrane protein LC3 (microtubule-associated protein 1 light chain 3) [4]. The phosphatidylinositol-3-phosphate-(PI3P-) binding protein Alfy (autophagy-linked FYVE domain containing protein) was also shown to be required for selective degradation of aggregated proteins such as polyQ [8, 9] although a LIR/LRS motif has not yet been identified in Alfy sequence.

**Figure 1:**
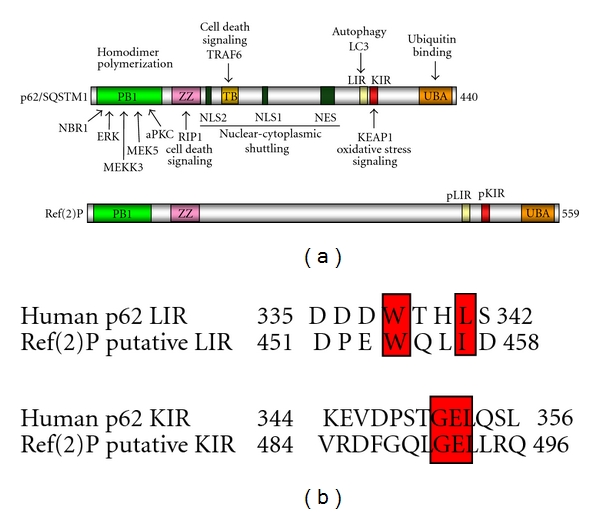
Schematic presentation of functional and structural domains of p62 and its *Drosophila* orthologue, Ref(2)P. (a) p62 consists of a PB1 domain (Phox and Bem1p domain) which is responsible for the interaction with the autophagy receptor NBR1 and the protein kinases ERK, MEKK3, MEK5, PKC*ζ*, and PKC*λ*/*ι*. The PB1 domain is followed by a ZZ-type zinc finger domain which contains the binding site for RIP1 and a TB domain which harbors the binding site of TRAF6. Nuclear localization signals (NLSs) and nuclear export signal (NES) are also present. p62 contains a LIR (LC3-interacting region) and a KIR (KEAP1-interacting region) motif and a C-terminal UBA (ubiquitin associated) domain responsible for binding to ubiquitin. Ref(2)P has similar structural and functional domains compared to p62. It consists of a PB1 domain which is followed by a ZZ-type zinc finger domain and a C-terminal UBA domain responsible for binding to ubiquitin. Ref(2)P also contains putative LIR and KIR motifs. (b) Bioinformatic prediction of Ref(2)P's putative LIR and KIR motifs and alignment with human p62's motifs. The functional roles of putative LIR and KIR motifs of Ref(2)P have to be tested experimentally.

Landmark studies from Johansen's group indicated that mammalian p62/SQSTM1 is degraded selectively by autophagy and introduced the significant role of p62/SQSTM1 in autophagy [10, 11]. p62/SQSTM1 is the first identified and most studied autophagy cargo receptor. It is a multifunctional scaffold protein that serves a large variety of cellular functions [4, 12, 13]. The human p62 protein is 440 amino acids long and contains several structural and functional motifs [4] (Figure 1(a)). A Phox and Bem1p domain (PB1 domain) is located at the N-terminus and is required for di- and multimerization of the protein as well as interaction with the protein kinases MEKK3, MEK5, ERK, PKC*ζ*, and PKC*λ*/*ι* and autophagy receptor NBR1 [4]. A zinc-finger-type (ZZ-type) domain follows the PB1 domain and is the binding site of receptor-interacting serine-threonine kinase 1 (RIP1) [12, 14]. Subsequently, there is a TRAF6-binding (TB) domain which contains the binding site of E3 ubiquitin-protein ligase TRAF6 [12, 14]. Nuclear-cytoplasmic shuttling of the protein is mediated by nuclear localization signals (NLSs) and nuclear export signal (NES) which are also present [15]. p62/SQSTM1 contains a LIR/LRS motif and a kelch-like ECH-associated protein 1 (KEAP1) interacting region (KIR) motif responsible for the interaction with LC3 and KEAP1, respectively [11, 16, 17]. The C-terminus of p62 harbors a ubiquitin-associated (UBA) domain required for its binding to mono- and polyubiquitin [4] (Figure 1(a)).

**Figure 2:**
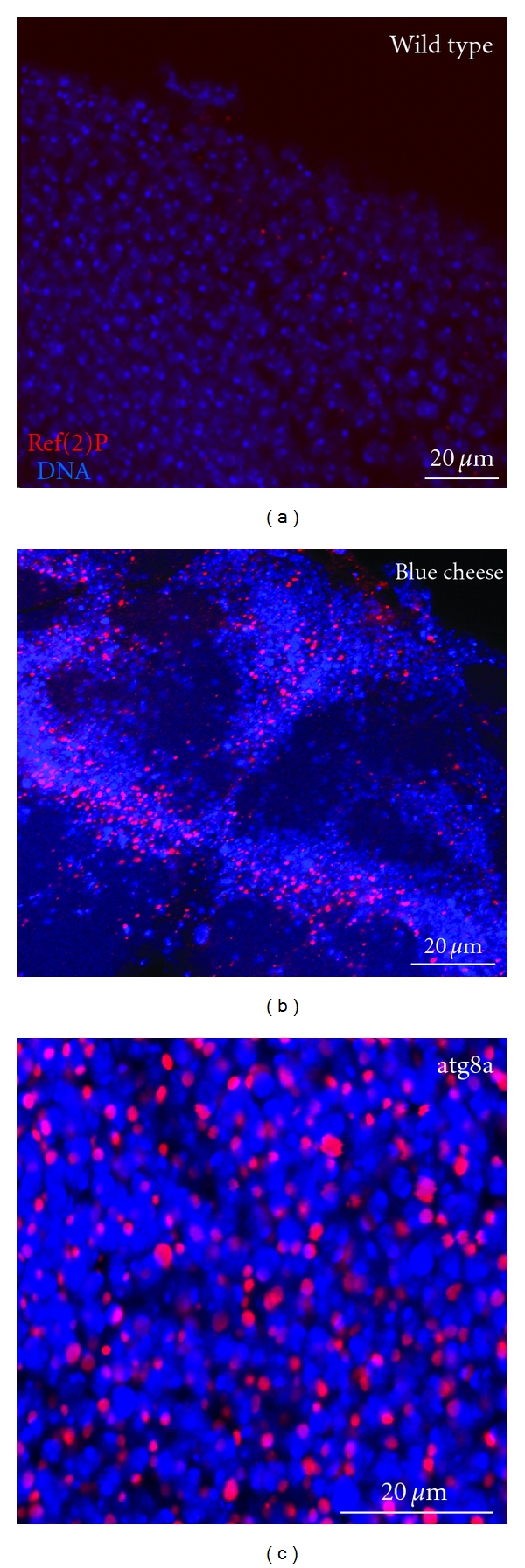
Ref(2)P accumulates in the adult brain of atg8a and blue cheese mutant flies. Confocal micrographs of superficial sections of the adult brain cortex of a wild-type fly (a), a blue cheese mutant fly (b), and an autophagy mutant fly (c). The tissues are stained for Ref(2)P (red) and DNA (blue). Ref(2)P accumulates ubiquitously into large sphere-shaped inclusion bodies/aggregates in blue cheese and autophagy mutants compared to wild type.

The *Drosophila* single p62 homologue, Ref(2)P (*refractory to Sigma P ref(2)P/CG10360*), has 599 amino acids and contains an N-terminal PB1 domain followed by a ZZ-type zinc finger domain and a C-terminal UBA domain (Figure 1(a)) [13, 18]. Although Ref(2)P has not been shown to be a selective autophagic substrate directly, several lines of evidence support this. First, it has been shown that Ref(2)P is a major component of protein aggregates in flies that are defective in autophagy, in flies that have impaired proteasomal function, in *Drosophila* models of human neurodegenerative diseases, and in protein aggregates formed during normal aging in *Drosophila* adult brain [18] (Figure 2). The abilities of Ref(2)P to oligo- and multimerize (through its PB1 domain) and to bind ubiquitinated proteins (through its UBA domain) were shown to be required during the *in vivo* formation of protein aggregates in the adult brain of *Drosophila* [18].

Second, bioinformatic analysis of the sequence of Ref(2)P reveals the presence of a putative LIR motif. The human p62 LIR motif is a 22 amino acid long sequence which contains an evolutionarily conserved motif of three acidic residues followed by a tryptophan (DDDW in p62) [4]. Johansen and Lamark implemented a sequence logo from 25 different LIR motifs from 21 different proteins that all have been tested for binding to ATG8 family proteins. They showed that the LIR motif seems to be eight amino acids long and proposed that the consensus LIR motif could be written as D/E-D/E-D/E-W/F/Y-X-X-L/I/V. It seems that there is a requirement for aromatic residues in the W-site (W/F/Y) and also a requirement for large, hydrophobic residues in the L-site (L/I/V) [4]. Bioinformatic analysis of Ref(2)P sequence reveals the presence of a putative LIR between amino acids 451–458 with a sequence DPEWQLID, which fits very well with the criteria for aromatic residues at W site (W) and hydrophobic residues at L site (I) (Figure 1(b)). Bioinformatic prediction also reveals the presence of a putative KIR motif spanning between the amino acids residues 484–496 (Figure 1(b)). The functional roles of putative LIR and KIR motifs of Ref(2)P have to be tested experimentally *in vitro* and *in vivo*. Taken together, the above information suggest that Ref(2)P is a selective autophagy cargo receptor in *Drosophila melanogaster*.

Ref(2)P was initially characterized in a screen for modifiers of sigma virus multiplication [19–21]. Sigma virus belongs to the family of rhabdoviruses which have two natural hosts, either insect and vertebrate or insect and plant [22]. Sigma virus is an atypical rhabdovirus, since there are no known plants or vertebrate hosts, and it only infects *Drosophila* [23]. Sigma virus is widespread in natural populations of *Drosophila*, and flies infected with the virus exhibit reduced viability of infected eggs and lower survival over winter [23–25]. ref(2)P is the best characterized locus among five host loci which are involved in the control of Sigma virus infection and multiplication, including ref(1)H, ref(2)P and ref(3)D [19, 26–28]. *Drosophila* flies in nature contain two types of alleles: the permissive alleles of ref(2)P which allow efficient sigma virus multiplication, and the restrictive alleles which reduce the replication of the virus [19, 23]. In flies having the permissive alleles, the probability of infection may reach 100%, whereas, in flies with restrictive alleles the infection rate drops to 0.01%, at least for some viral strains [23, 28]. It appears that the restrictive allele appeared several thousands of years ago and spread in the population as a result of natural selection since it confers a selective advantage [29]. The appearance of the sigma virus strain capable of infecting *Drosophila* flies carrying the restrictive ref(2)P alleles occurred much more recently (25 years ago) and rapidly spread in natural population across Europe [30]. Homozygous Ref(2)P null flies are fully viable but the males are sterile. The molecular mechanisms of male sterility are not clear [19, 20]. Electron microscopy studies revealed that in the testes of ref(2)P^od1^ and ref(2)P^od3^ loss-of-function mutants (where Ref(2)P protein lacks the UBA domain) and ref(2)P^od2^ loss-of-function mutant (where Ref(2)P protein lacks the PB1 domain), characteristics of degeneration were frequently observed, such as the appearance of large myelin figures around the spermatids [20]. Additionally, the most striking difference was observed in the mitochondria, which varied in size and appeared degenerated [20]. Mammalian p62 has been shown to contribute to autophagic degradation of ubiquitinated mitochondria and to their clustering [31]. Therefore, it would be interesting to test this scenario in Ref(2)P mutant testis.

**Figure 3:**
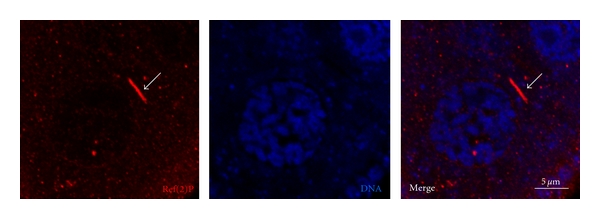
Ref(2)P localization in *Drosophila* egg chamber. Confocal micrograph of a middle section of a stage 8 egg chamber of wild-type fly, illustrating a portion of a nurse cell. The tissue is stained for Ref(2)P (red) and DNA (blue). Note the rod-like structure stained for Ref(2)P (arrow).

One open question is how Ref(2)P controls sigma virus multiplication at the molecular and cellular level. Work from Contamine's group suggests a direct interaction between Ref(2)P and a sigma virus protein, since Ref(2)P has been shown to interact with the sigma virus capsid P protein and to share conformation-dependent epitopes with the capsid N protein [32]. Additionally, Ref(2)P has been shown to interact genetically with DaPKC and the *Drosophila *homologue of TRAF6, dTRAF2, to participate in the Toll-signaling pathway, and to regulate the NF-*κ*B proteins Dorsal and DIF [33, 34]. Interestingly, mammalian p62 was shown to interact with sindbis virus capsid protein, and genetic knockdown of p62 blocked the targeting of viral capsid to autophagosomes [35]. Taken together, these results suggest that Ref(2)P may target sigma virus capsid for autophagosomal degradation and also may function as a scaffolding protein during assembly of viral protein complexes. This scenario has to be tested experimentally. Intriguingly, Ref(2)P was shown to accumulate in rod-shaped structures in *Drosophila* egg chambers, structures that may represent aggregates of viruses or bacteria (Figure 3).

 Another aspect of Ref(2)P function was recently reported in *Drosophila* hemocytes. Interestingly, Ref(2)P was shown to have a role in hemocyte spreading and protrusion formation [36]. This suggests that selective autophagy of an ubiquitinated substrate may function in an autophagy-dependent mechanism for cortical remodeling of hemocytes. Taken together, all the above information demonstrates that Ref(2)P, like its mammalian homologue p62, has diverse cellular functions whose molecular mechanisms have to be examined in detail.

#### 2.1.2. Blue Cheese, the *Drosophila* Homologue of the Mammalian Selective Aggregate Clearance Mediator Alfy

The mammalian phosphatidylinositol-3-phosphate-(PI3P-) binding protein Alfy was shown to be required for selective degradation of protein aggregates [8, 9, 37]. Alfy is a huge protein containing 3527 amino acids residues. It harbors several functional domains in the C terminus: a BEACH domain followed by a series of WD40 repeats and a PI(3)P-binding FYVE domain [8]. Despite its FYVE-domain which would suggest a localization to PI(3)P-rich endosomes, Alfy is not found on endosomes but instead localizes mainly to the nuclear envelope. Under conditions of starvation or proteasomal inhibition, Alfy relocalizes to cytoplasmic structures located close to autophagic membranes and ubiquitin-containing protein aggregates. Electron microscopy studies revealed that similar structures can be found within autophagosomes [8]. Importantly, Alfy was shown to be required for selective degradation of aggregated proteins such as polyQ-cotaining mutant huntingtin [9]. This function was proposed to be mediated by Alfy's physical interaction with PI(3)P, Atg5, and p62 [9, 37]. Therefore, Alfy functions as a scaffold receptor for recruitment of misfolded, ubiquitinated proteins to the autophagosomal membrane that become degraded by autophagy.

Blue cheese is the *Drosophila* homologue of Alfy and is highly conserved with its human homologue (~50% identity between fly and human homologs) [8, 38], and it contains similar functional domains at its C-terminal. *blue cheese* mutant flies exhibit a reduced adult life span and age-related neurodegeneration associated with accumulation of ubiquitin-conjugated protein aggregates throughout the adult central neruous system, neural atrophy, and cell death [38]. Ref(2)P accumulates in ubiquitinated inclusions in the brain of *blue cheese* mutant flies, suggesting that blue cheese is required for autophagic degradation of p62-associated ubiquitinated proteins *in vivo* [38] (Figure 2).

Finley and colleagues performed a genetic modifier screen for blue cheese genetic interactions based on alteration of the blue cheese eye phenotype.They found that recessive mutations in lysosomal trafficking genes and members of the ubiquitin and SUMO signaling pathways as well as in cytoskeletal and motor proteins have potential genetic interactions with Blue cheese [39]. They also showed that mutations of several lysosomal transport genes also alter high-molecular-weight UB-protein profiles and reduce adult life span [39]. Importantly, it was recently shown by Simonsen and Finley groups that overexpression of the C-terminal region of Blue cheese ameliorates neurodegeneation related phenotypes *in vivo* [9]. The authors tested the enhanced expression of Blue cheese in *Drosophila* eye model of polyglutamine toxicity, where UAS-polyQ127 transgene was expressed in the fly eye. It is well established that poly Q expression in the eye results in ommatidial disorganization, pigmentation loss, reduced eye size, and the appearance of necrotic regions. Enhanced expression of full-length Blue cheese (UAS-FL-Bchs) or C-terminal Blue cheese (UAS-bchs-C1000) with UAS-polyQ127 in the eye resulted in reduced number of necrotic areas and an overall improvement in eye size, morphology, and pigmentation. Taken together, these results suggest that the Alfy/Bchs proteins have a role in macroautophagic clearance of aggregation-prone proteins.

### 2.2. Mitophagy, Xenophagy, and Nucleophagy in *Drosophila*

Selective autophagy was recently shown to play an important role in the quality control of organelles and intracellular pathogens [31, 40]. However, mitophagy (selective autophagy of mitochondria), xenophagy (selective autophagy of bacteria and viruses), and nucleophagy (selective autophagy of nuclear fragments) are largely unexplored in *Drosophila*. Moreover, pexophagy (selective autophagy of peroxisomes) is not described yet in *Drosophila*. In the following lines, I will summarize what is reported so far in the literature about the processes above in *Drosophila*.

#### 2.2.1. Mitophagy

Mitophagy has been recently described in yeast and mammals [31]. In yeast, the outer mitochondrial membrane protein Atg32 binds to the autophagosomal membrane protein Atg8 through its LIR motif [41]. In mammals, mitophagy was described during the physiological process of red blood cell differentiation and it requires the outer mitochondrial membrane protein NIP3-like protein NIX, which is also binds to LC3 through its LIR motif [42, 43]. Additionally, when mitochondria are damaged and depolarized, the kinase PTEN-induced putative kinase protein 1 (PINK1) accumulates to mitochondria and recruits the E3 ubiquitin ligase Parkin from the cytoplasm specifically to the damaged mitochondria. Subsequently, Parkin ubiquitylates mitochondrial proteins and promotes mitochondrial degradation by autophagy [31].

Genetic studies in *Drosophila* showed that the PINK1-Parkin pathway promotes mitochondrial fission or alternatively inhibit their fusion [44, 45]. It was recently shown in S2 cells that *Drosophila* PINK1 localizes to depolarized mitochondria and recruits Parkin and this promotes mitochondria degradation by autophagy [46]. Importantly, the profusion factor mitofusin (Mfn; also known as marf in *Drosophila*) was shown to be a novel substrate of Parkin [46]. Interestingly, it was also reported that activation of autophagy through Atg1 overexpression rescues PINK1 mutant phenotypes in *Drosophila* [47]. These studies suggest that, like in mammals, mitophagy also occurs in *Drosophila* and is dependent on PINK1 and Parkin, although the molecular details have to be further clarified.

Finally, it was recently reported that mitochondrial dynamics are abnormal in autophagy deficient egg chamber [48]. Dying atg1 germline mutant egg chambers exhibit abnormal mitochondrial remodeling that included the presence of mitochondrial islands suggesting that there is a cross-talk between autophagy, mitochondrial dynamics, and cell death during *Drosophila* oogenesis [48].

#### 2.2.2. Xenophagy

Autophagy has been associated with the elimination of intracellular pathogens during mammalian innate immune responses, a process called xenophagy [40]. In *Drosophila,* xenophagy is largely unexplored. There are two reports that provide evidence for conserved mechanisms of xenophagy in *Drosophila*. In the first one, Kurata and colleagues reported that, in primary *Drosophila* hemocytes and S2 cells, autophagy prevented the intracellular growth of *Listeria monocytogenes* and promoted host survival after this infection [49]. Additionally, recognition of diaminopimelic acid-type peptidoglycan by the pattern-recognition receptor PGRP-LE was required for the induction of autophagy. Importantly, autophagy induction occurred independently of the Toll and IMD innate-signaling pathways [49].

In a second study, it was found that autophagy implements an antiviral role against the mammalian viral pathogen vesicular stomatitis virus (VSV) in *Drosophila *S2 cells as well as in adult flies [50]. The surface glycoprotein of VSV, VSVG, was shown to be the pathogen-associated molecular pattern that initiates the autophagic response. Autophagy was shown to restrain viral replication, and repression of autophagy resulted in increased viral replication and pathogenesis. Importantly, it was shown that this response was regulated by the phosphatidylinositol 3-kinase (PI3K)/Akt signaling pathway which controls autophagy in response to nutrient availability [50]. These data suggest that xenophagy occurs in *Drosophila,* and the molecular mechanisms are well conserved compared to mammals.

#### 2.2.3. Nucleophagy

Nucleophagy is the process where parts of the nucleus can be specifically degraded by autophagy [51]. Nucleophagy is best characterized in yeast *Saccharomyces cerevisiae*, and is called piecemeal microautophagy [52]. During piecemeal microautophagy of the nucleus there is formation of nucleus-vacuole junctions where parts of the nucleus are sequestered into invaginations of the vacuolar membrane, followed by fission of nuclear fragments, and its release into the vacuolar lumen, where they are degraded. A direct interaction of the nuclear membrane protein Nvj1p with that vacuole protein Vac8p of the vacuole are required for this process [51, 52]. Recently, nucleophagy was also reported in mammals in nuclear envelopathies caused by mutations in the genes encoding A-type lamins (LMNA) and emerin (EMD) [53]. Nucleophagy was also observed rarely in wild-type cells [53].

In *Drosophila,* nuclear autophagy has been recently described during the cell death of nurse cells in late oogenesis [54]. Immunofluorescence analysis of mCherry-DrAtg8a autophagy marker in the nurse cells during the late stages of oogenesis revealed the presence of large autolysosomes adjacent to or attached to the condensed and fragmented nurse cell nucleus. Ultrastructural analysis revealed the presence of large autolysosomes which contained condensed material resembling the material of the fragmented nurse cell nucleus, suggesting that the nurse cell nuclear fragments are removed by autophagy [54].

### 2.3. Selective Degradation of Proteins in *Drosophila*

Autophagy has been shown to be responsible for the selective degradation of proteins in mammals and yeast like beta-synuclein [55], catalase [56], and acetaldehyde dehydrogenase [57]. In *Drosophila,* there is also a growing number of cases in which proteins can be preferentially degraded by autophagy.

#### 2.3.1. Degradation of Survival Factors

Degradation of survival factors is a way of cell to die [58]. There are two recent reports that support this hypothesis in *Drosophila*. In the first study, we have demonstrated that the inhibitor of apoptosis protein dBruce was degraded by autophagy in the nurse cells during cell death in late oogenesis [54]. Genetic inhibition of autophagy in the female germline resulted in late stage egg chambers containing persistent nurse cell nuclei that did not contain fragmented DNA and in attenuation of caspase-3 activation. Importantly, we found that *Drosophila* inhibitor of apoptosis dBruce is degraded by autophagy, and this is responsible to control DNA fragmentation [54]. A second report showed that degradation of inhibitor of apoptosis protein DIAP1 during developmental dendrite pruning of *Drosophila* class IV dendritic arborization neurons is depended on Valosin-containing protein (VCP), a ubiquitin-selective AAA chaperone involved in endoplasmic reticulum-associated degradation and the maturation of autophagosomes [59, 60]. These results suggest that autophagic degradation of survival factors can cause cell death during development in *Drosophila*.

#### 2.3.2. Degradation of Rhodopsin and Retinal Degeneration

Activated rhodopsin is degraded in endosomal pathways in normal photoreceptor cells in *Drosophila,* and accumulation of activated rhodopsin in some *Drosophila *mutants leads to retinal degeneration [61]. In a recent study, it was reported that activated rhodopsin is degraded by autophagy in order to prevent retinal degeneration [62]. Light-dependent retinal degeneration in the *Drosophila *eye is caused by silencing or mutation of autophagy genes, such as autophagy-related protein 7 and 8, or genes essential for PE (phosphatidylethanolamine) biogenesis and autophagosome formation, including phosphatidylserine decarboxylase (Psd) and CDP-ethanolamine:diacylglycerol ethanolaminephosphotransferase (Ept). Silencing of atg-7/8 or Psd/Ept resulted in an increase in the amount of rhodopsin localized to Rab7-positive late endosomes [62]. These results suggest that autophagic and endosomal/lysosomal pathways suppress light-dependent retinal degeneration and that rhodopsin is a substrate for autophagic degradation in this context.

#### 2.3.3. Degradation of Highwire

Beyond its role in cellular homeostasis, autophagy is implicated in the regulation of developmental growth and remodeling of various cells and tissues during development [63]. One such example in *Drosophila* is the synaptic development of the larval neuromuscular junction. Shen and Ganetzky showed that autophagy promotes the synaptic development of the *Drosophila* larval neuromuscular junction, by downregulating an E3 ubiquitin ligase, Highwire, which restrains neuromuscular junction growth via a MAPKKK pathway [64, 65]. Autophagy mutants exhibit neuromuscular junction undergrowth and Atg1 overexpression, resulting in neuromuscular junction overgrowth. Moreover, overgrowth associated with Atg1 overexpression is suppressed by mutations in *atg18*, demonstrating that this overgrowth is due to elevated levels of autophagy [64, 65]. In a recent paper, *Drosophila *Rae1 was identified as a component of the Highwire complex. Loss of *Rae1 *function in neurons results in morphological defects at the neuromuscular junction that are similar to those seen in Highwire mutants [66]. The authors found that Rae1 physically and genetically interacts with Highwire and limits synaptic terminal growth by regulating the MAP kinase kinase kinase Wallenda. Moreover, they found that the Rae1 is sufficient to promote Highwire protein abundance by binding to Highwire and protecting it from autophagic degradation [66]. Together, these findings indicate that Rae1 prevents autophagy-mediated degradation of Highwire and that selectively controls Highwire protein abundance during synaptic development.

## 3. Concluding Remarks and Future Directions

From the literature analyzed above, it is obvious that the molecular mechanisms of selective autophagy in *Drosophila* remain largely unexplored. The precise mechanisms of selective autophagy of organelles and proteins has not been directly shown in *Drosophila*, and the molecular details of the interaction of selective autophagy receptors Ref(2)P and blue cheese with the autophagic machinery have to be shown experimentally. The presence of putative LIR motif in Ref(2)P offers a fertile ground for further functional analysis *in vivo*. p62 and Ref(2)P have been proposed to collect ubiquitinated proteins and target them for degradation [4]. It would therefore be interesting to test whether induced expression of Ref(2)P ameliorates phenotypes related to neurodegeneration *in vivo*. It will also be important to elucidate in details how small or large aggregates are removed per se. Elucidation of these processes may have applications in fighting aggregation-related diseases, such as neurodegenerative diseases as well as cancer. There is emerging evidence that mammalian p62 directly interacts with Keap1 and that p62 is a target gene for Nrf2 transcription factor implicated in oxidative stress signaling [4, 13]. It would be interesting to test the interaction of Ref(2)P with the *Drosophila* homologue of Keap1, dkeap1 [67]. It would also be interesting to test whether the *Drosophila* homologues of BNIP3-like proteins play a role in selective degradation of mitochondria.

In conclusion, *Drosophila* offers a fertile ground for studying the molecular mechanisms of selective autophagy. Future studies will hopefully uncover the molecular details of this process.
